# Magnolol Protects against MPTP/MPP^**+**^-Induced Toxicity via Inhibition of Oxidative Stress in *In Vivo* and *In Vitro* Models of Parkinson's Disease

**DOI:** 10.1155/2012/985157

**Published:** 2012-05-08

**Authors:** Akiko Muroyama, Aya Fujita, Cheng Lv, Shota Kobayashi, Yoshiyasu Fukuyama, Yasuhide Mitsumoto

**Affiliations:** ^1^Laboratory of Alternative Medicine and Experimental Therapeutics, Department of Clinical Pharmacy, Faculty of Pharmaceutical Sciences, Hokuriku University, Kanazawa, Ishikawa 920-1181, Japan; ^2^Faculty of Pharmaceutical Sciences, Tokushima Bunri University, Tokushima 770-8514, Japan

## Abstract

The aim of this study is to investigate the role of magnolol in preventing 1-methyl-4-phenyl-1,2,3,6-tetrahydropyridine (MPTP-) induced neurodegeneration in mice and 1-methyl-4-phenylpyridinium ion-(MPP^+^-) induced cytotoxicity to human neuroblastoma SH-SY5Y cells and to examine the possible mechanisms. Magnolol (30 mg/kg) was orally administered to C57BL/6N mice once a day for 4 or 5 days either before or after MPTP treatment. Western blot analysis revealed that MPTP injections substantially decreased protein levels of dopamine transporter (DAT) and tyrosine hydroxylase (TH) and increased glial fibrillary acidic protein (GFAP) levels in the striatum. Both treatments with magnolol significantly attenuated MPTP-induced decrease in DAT and TH protein levels in the striatum. However, these treatments did not affect MPTP-induced increase in GFAP levels. Moreover, oral administration of magnolol almost completely prevented MPTP-induced lipid peroxidation in the striatum. In human neuroblastoma SH-SY5Y cells, magnolol significantly attenuated MPP^+^-induced cytotoxicity and the production of reactive oxygen species. These results suggest that magnolol has protective effects via an antioxidative mechanism in both *in vivo* and *in vitro* models of Parkinson's disease.

## 1. Introduction

Parkinson's disease (PD) is a progressive neurodegenerative disorder characterized by the selective loss of nigral dopaminergic neurons resulting in reduced striatal dopamine and the cardinal clinical features such as bradykinesia, resting tremor, rigidity, and postural instability [1]. Currently, pharmacotherapy and surgical approaches for the treatments of PD can only improve the neurological symptoms [2]. Furthermore, long-term treatment with the dopamine precursor levodopa often leads to the development of debilitating dyskinesias [2]. Therefore, to search neuroprotective therapies using pharmacological and nonpharmacological approaches could be important to delay the progression of pathogenesis in PD. The cause of PD remains unknown, but a valuable clue has been suggested by discovery of 1-methyl-4-phenyl-1,2,3,6-tetrahydropyridine (MPTP; [3]). MPTP selectively damages the dopaminergic pathways in a pattern similar to that seen in PD and induces a parkinsonian syndrome in humans, monkeys, and mice [1, 4, 5]. The discovery that MPTP acts through inhibition of complex I of the electron transport chain stimulated study of mitochondrial function in the brains from patients with PD [6, 7]. Mizuno et al. [8] proposed that energy crisis is the most important mechanism of nigral cell death in PD. In addition, oxidative stress has also been implicated as an important contributor to nigral cell death in PD [9, 10], but it is a secondary phenomenon on respiratory failure, because respiratory failure will increase oxygen-free radical and consume glutathione [11, 12]. Oxidative stress and mitochondrial failure produce a vicious cycle in nigral neurons. 

**Figure 1:**
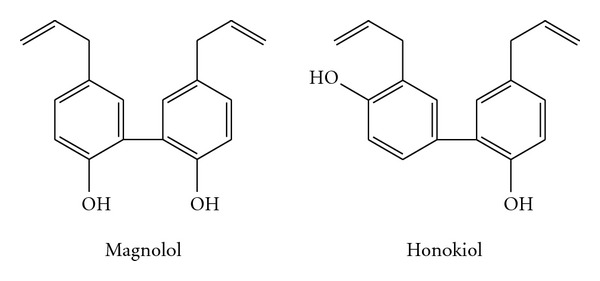
Structures of magnolol and honokiol.

Magnolol and honokiol are the main constituents of the stem bark of *Magnolia obovata* Thunb and *Magnolia officinalis* Rhed [13] (Figure 1). They have been used as traditional medicines in China and Japan and have a wide spectrum of pharmacological activity [14]. In the central nervous system, these compounds exhibit anxiolytic and antidepressant-like effects when taken orally [15, 16]. Moreover, magnolol and honokiol have neuroprotective effects on the central nervous system using both *in vivo* and *in vitro* models [17–21]. Honokiol reduces infarct brain areas after cerebral ischaemia of mice associated with suppression of reactive oxygen species (ROS) production and preservation of mitochondrial functions through its antioxidative properties [19]. In cultured rat cortical neurons, honokiol and magnolol show neurotrophic and neuroprotective effects associated with activation of the intracellular signal transduction cascade [22, 23]. Furthermore, magnolol and honokiol prevent age-related learning and memory impairment by preserving cholinergic neurons in the forebrain in senescence-accelerated prone mice (SAMP; [20]). Recently, it has been reported that magnolol prevents the loss of dopaminergic neurons in 6-hydroxydopamine- (6-OHDA-) treated PD mouse model [21].

In the present study, we demonstrated that magnolol prevents MPTP-induced neurodegeneration in the mouse model of PD and 1-methyl-4-phenylpyridinium ion-(MPP^+^-) induced cytotoxicity to human neuroblastoma SH-SY5Y cells. Furthermore, we found that its neuroprotective effect against these toxins could be associated with the attenuating the oxidative stress in both *in vivo* and *in vitro *studies.

## 2. Materials and Methods

### 2.1. Animals

Male C57BL/6 N mice (9–12 weeks, Charles River Japan, Atsugi, Japan) were used. They were housed at an ambient temperature of 23 ± 2°C under a 12 h light/12 h dark cycle (lights on, 7:00 AM) with free access to food and water. All procedures were performed in accordance with the Guidelines for Animal Care and Use in Hokuriku University.

### 2.2. Drug Preparation and Treatment

MPTP hydrochloride (Sigma-Aldrich, MO, USA) was dissolved in saline and administered intraperitoneally (i.p.). Magnolol was isolated from methanol extract of the *M. officinalis* Rhed. The purity was determined by high performance liquid chromatography (single peak) and by nuclear magnetic resonance spectra [24]. It was dispersed in a saline suspension containing 5% Gum arabic and orally administered using an oral-zonde needle. In the pre-MPTP treatment with magnolol, the mice (3-4 per group) received magnolol (30 mg/kg) for 5 consecutive days and were injected MPTP (20 mg/kg every 2 h, four times) [25] at the day after the last magnolol administration. Control mice were administered those vehicles. The mice were killed at 3 days after the MPTP treatment. In the post-MPTP treatment with magnolol, the mice (3-4 per group) received a single injection of MPTP (40 mg/kg), and then magnolol (30 mg/kg/day) was administered at the day just after MPTP treatment and given daily for 4 consecutive days. The mice were killed at 1 h after the last magnolol administration.

### 2.3. Western Blot Analysis

Western blot analysis was performed as described previously [25]. The striatal tissues were homogenized in ice-cold homogenization buffer (0.32 M sucrose containing 4 *μ*g/mL pepstatin, 5 *μ*g/mL aprotinin, 20 *μ*g/mL trypsin inhibitor, 4 *μ*g/mL leupeptin, 0.2 mM PMSF, 2 mM EDTA, 2 mM EGTA, and 20 mM Hepes, at pH 7.2) using a microtube homogenizer. Protein concentrations were determined using BCA Protein Assay Kit (Pierce, IL, USA). The homogenates were solubilized with Laemmli sample buffer and subjected to 10% sodium dodecyl sulphated-polyacrylamide gel electrophoresis at 20 *μ*g protein per lane, and then transferred onto polyvinylidene difluoride (PVDF) membranes (Millipore, MA, USA). Blots were incubated with rat monoclonal antibody against the dopamine transporter (DAT, MAB369, Chemicon, CA, U.S.A.), rabbit polyclonal antibody against the tyrosine hydroxylase (TH, AB152, Chemicon) and mouse monoclonal antibody against the glial fibrillary acidic protein (GFAP, Sigma-Aldrich, MO, U.S.A.). The mouse monoclonal antibody against the actin (MAB1501, Chemicon) was used as a loading control. The membranes were incubated with alkaline phosphatase-conjugated secondary antibody, and then developed in accordance with the manufacturer's instructions. The densities of immunoreactive bands were analyzed by image analysis software (Image J 1.36, NIH). Densitometric analysis was performed to quantify relative protein level against actin protein.

### 2.4. Measurement of Lipid Peroxidation

Lipid peroxidation was assessed by determining the concentrations of thiobarbituric acid reactive substances (TBARS). The TBARS determination was performed as described previously with a minor modification [26]. The striatal tissue was homogenized in homogenization buffer without sucrose, and treated with SDS (8%, w/v), acetic acid (20%), and thiobarbituric acid (0.8%). The resulting mixture was incubated at 95°C for 60 min. After cooling at room temperature, 2.5 mL of *n*-butanol and pyridine (15 : 1) was added, and the mixture was shaken vigorously. After centrifugation at 1,600 ×g for 10 min, the absorbance of the supernatant (organic layer) was measured at 532 nm using a Fluorescence Spectrophotometer F-4500 (Hitachi Koki Co., Ltd.).

### 2.5. Cell Culture and Drug Preparation

Human neuroblastoma SH-SY5Y cells were obtained from Otsuka Pharmaceutical Co. Ltd. (Tokushima, Japan). Cultures were maintained in Dulbecco's modified Eagle's medium (DMEM) supplemented with 10% heat-inactivated fetal bovine serum, 100 U/mL penicillin, 100 *μ*g/mL streptomycin at 37°C under a humidified atmosphere of 95% air and 5% CO_2_.

MPP^+^ iodide (Sigma-Aldrich, MO, USA) was dissolved in the treatment medium, serum-free DMEM plus 1% N2-supplement (Invitrogen Corp., CA, USA). A stock solution of magnolol was prepared in dimethylsulfoxide (DMSO; final concentration 0.1%) and dissolved in same medium.

### 2.6. Alamar Blue Assay

Mitochondrial oxidation-reduction (REDOX) activity was estimated using Alamar blue dye (Invitrogen Corp., CA, USA). Alamar blue is a fluorescent indicator that is intermediate only between final reduction of molecular oxygen and cytochrome oxidase. Cells were seeded in 96-well plates at the density of 1.0 × 10^5^ cells/cm^2^ and cultured in the growth medium for 48 h. The medium was changed to serum-free DMEM plus 1% N2-supplement containing MPP^+^ and/or magnolol. At 24 h after treatment, cells were incubated with Locke's buffer (154 mM NaCl, 5.6 mM KCl, 2.3 mM CaCl_2_, 1.0 mM MgCl_2_, 3.6 mM NaHCO_3_, 5 mM glucose, 5 mM Hepes, at pH 7.2) containing 10% Alamar blue dye for 2 h at 37°C. Alamar blue fluorescence was measured using a fluorometric plate reader (Fluoroskan Ascent, Thermo Fisher Scientific K.K., Vantaa, Finland) at 544 nm excitation and 590 nm emission.

### 2.7. Measurement of Intracellular ROS Production

Intracellular ROS production was measured by using a ROS-sensitive fluorescent dye, 2′7′-dichlorodihydrofluorescein diacetate (H_2_DCF-DA, Invitrogen Corp., CA, USA). SH-SY5Y cells were seeded in 96-well plates at the density of 1.5 × 10^5^ cells/cm^2^ and cultured in the growth medium for 48 h. Cells were loaded with serum-free DMEM plus 1% N2-supplement containing 10 *μ*M H_2_DCF-DA for 30 min at 37°C, and then MPP^+^ and/or magnolol were treated for 24 h. The fluorescent intensity of 2′7′-dichlorofluorescein (DCF) was measured using a plate reader at 485 nm excitation and 538 nm emission.

### 2.8. Statistical Analysis

The statistical significance of the differences with absolute values was assessed by one-way ANOVA followed by Tukey test or Dunnett's test. The analyses were performed with the statistical analysis system StatMate III (ATMS Co., Ltd., Tokyo, Japan). A probability value of less than 5% was considered statistically significant.

## 3. Results

### 3.1. Effect of Pre- and Post-MPTP Treatment with Magnolol on Dopaminergic Neurotoxicity in C57BL/6 Mice

**Figure 2:**
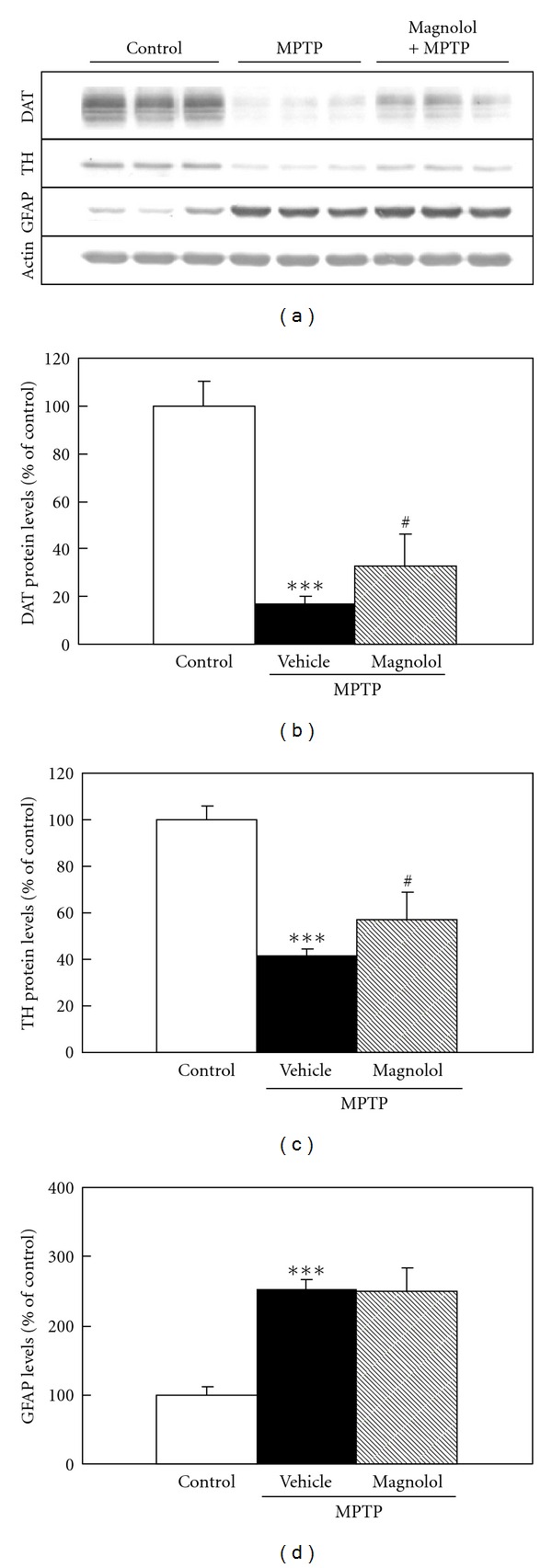
Effect of pre-MPTP treatment with magnolol on DAT, TH and GFAP protein levels in the striatum of C57BL/6 mice. Mice were administered daily with magnolol (30 mg/kg) for 5 days, and received 4 injections of 20 mg/kg MPTP at 2 h intervals at the day after the last magnolol treatment. The evaluations were performed at 3 days after MPTP treatment. Representative immunoblots of DAT, TH and GFAP protein in the striatum are shown (a). Actin protein was used as housekeeping protein. Immunoblots of DAT (b), TH (c), and GFAP (d) in the striatum were quantified in each group. Densitometric analysis of protein bands were performed using software (Image J 1.36, NIH). The data were expressed as % of control. Results are means ± SD (*n* = 6-7) from two independent experiments. Statistical comparisons were carried out by one-way ANOVA followed by Scheffe test. ***P < 0.001 versus control, ^#^ *P* < 0.05 versus MPTP + vehicle.

**Figure 3:**
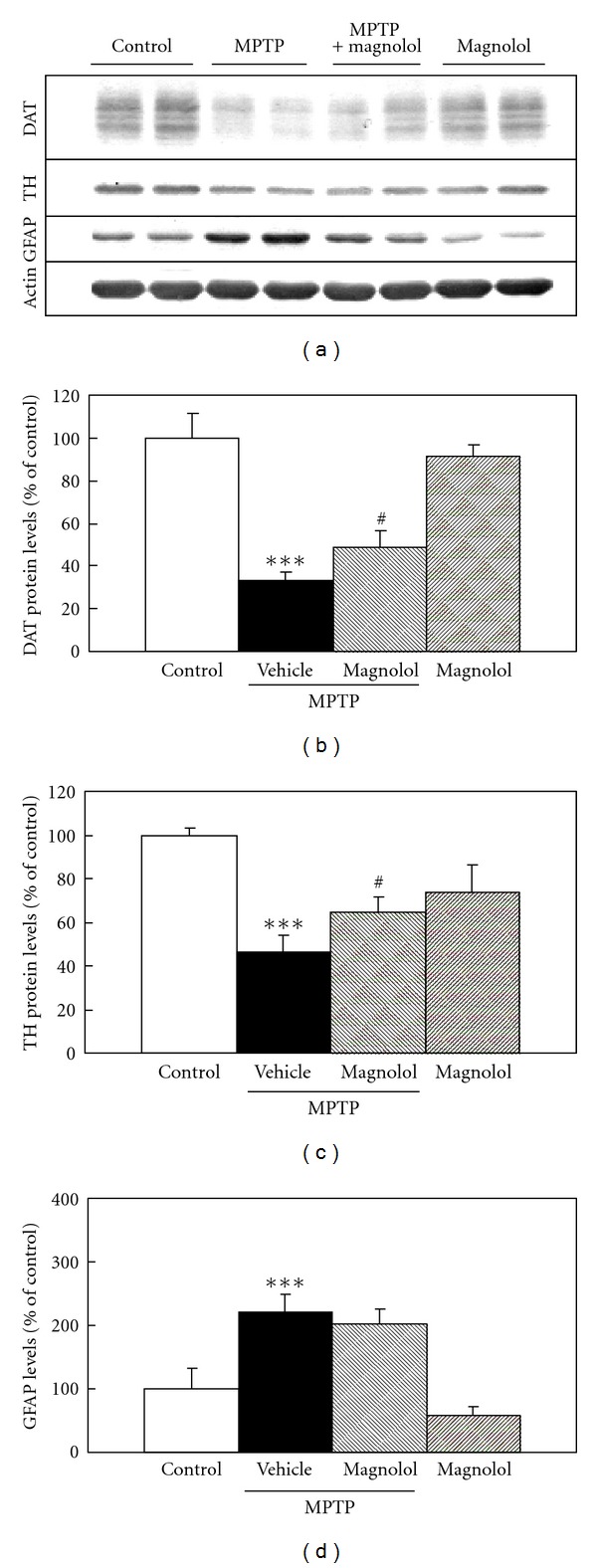
Effect of post-MPTP treatment with magnolol on DAT, TH, and GFAP protein levels in the striatum of C57BL/6 mice. Mice were received a single injection of 40 mg/kg MPTP and administered daily with magnolol (30 mg/kg) for 4 days at the day just after MPTP treatment. The evaluations were performed at 1 h after the last treatment with magnolol. Representative immunoblots of DAT, TH, and GFAP protein in the striatum are shown (a). Actin protein was used as house keeping protein. Immunoblots of DAT (b), TH (c), and GFAP (d) in the striatum were quantified in each group. Densitometric analysis of protein bands was performed using software (Image J 1.36, NIH). The data were expressed as % of control. Results are means ± SD (*n* = 3-4) from three independent experiments. Statistical comparisons were carried out by one-way ANOVA followed by Tukey test. ***P < 0.001 versus control, ^#^ *P* < 0.05 versus MPTP + vehicle.

We evaluated the effect of pre- and post-MPTP treatment with magnolol on striatal dopaminergic neurons in mice. In the pre-MPTP treatment with magnolol, mice were administered daily with magnolol (30 mg/kg) for 5 days and received 4 injections of 20 mg/kg MPTP at 2 h intervals at the day after the last treatment with magnolol (Figure 2). Protein levels of DAT, TH, and GFAP were changed to 17%, 41% and 250% of control by MPTP treatment, respectively (Figures 2(b), 2(c), and 2(d)). Pre-MPTP treatment with magnolol significantly attenuated MPTP-induced decrease in DAT and TH protein levels to 33% and 57% of control, respectively (Figures 2(b) and 2(c)), whereas the increase in GFAP levels were not affected (Figure 2(d)). In the post-MPTP treatment with magnolol, mice were received a single injection of 40 mg/kg MPTP and administered daily with magnolol (30 mg/kg) for 4 days at the day just after MPTP treatment (Figure 3). Protein levels of DAT, TH, and GFAP were changed to 33%, 47%, and 220% of control by MPTP injection, respectively (Figures 3(b), 3(c), and 3(d)). Post-MPTP treatment with magnolol significantly attenuated the MPTP-induced decrease in DAT and TH protein levels to 48% and 64% of control, respectively (Figures 3(b) and 3(c)), whereas the increase in GFAP levels was not affected (Figure 3(d)). Magnolol alone did not significantly affect these protein levels.

### 3.2. Effect of Magnolol on Lipid Peroxidation in MPTP-Treated Mice

**Figure 4:**
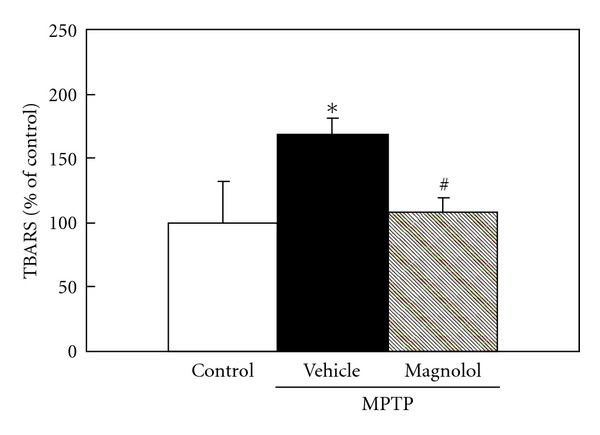
Effect of magnolol on lipid peroxidation levels in the striatum of MPTP-treated C57BL/6 mice. Mice were received a single injection of 30 mg/kg MPTP and administered with magnolol (30 mg/kg) at the day just after MPTP treatment. At 6 h after MPTP treatment, the lipid peroxidation products were measured as TBARS in the striatum. These are representative results from three independent experiments. The data were expressed as % of control. Results are means ± SD (*n* = 3). Statistical comparisons were carried out by one-way ANOVA followed by Tukey test. *P < 0.05 versus control, ^#^ *P* < 0.05 versus MPTP + vehicle.

To determine the mechanism whereby magnolol prevent loss of dopaminergic neurons, we examined the effect of magnolol on MPTP-induced lipid peroxidation in the striatum (Figure 4). The level of TBARS is an index of lipid peroxidation and indicated as markers of oxidative stress status in the MPTP mouse model [26]. Mice were received a single injection of 30 mg/kg MPTP and administered with magnolol (30 mg/kg) at the day just after MPTP treatment. TBARS level increased to 168% of control level in the striatum at 6 h after MPTP injection. Oral administration of magnolol almost completely inhibited the MPTP-induced increase in TBARS levels.

### 3.3. Effect of Magnolol on MPP^+^-Induced Cytotoxicity in Human Neuroblastoma SH-SY5Y Cells

**Figure 5:**
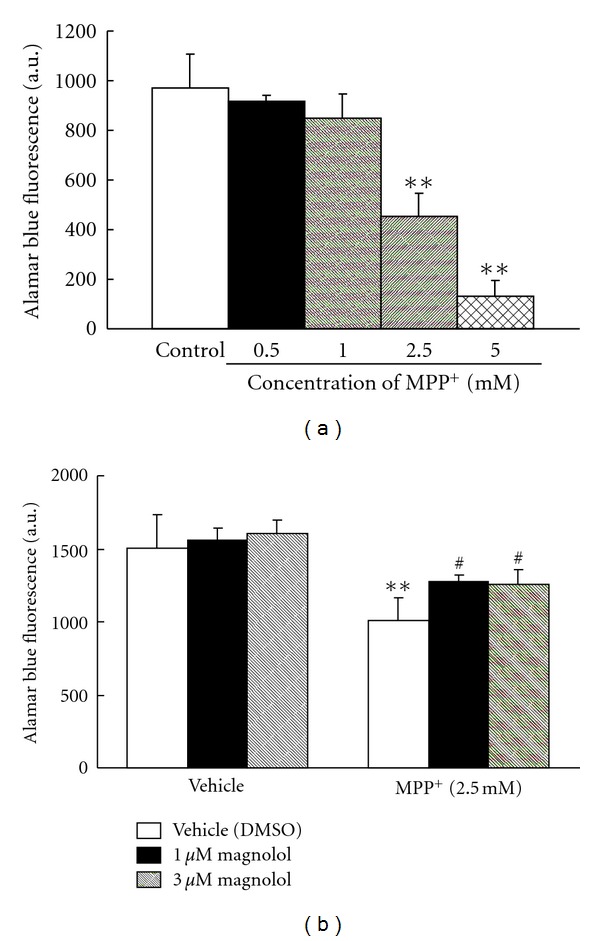
Effect of MPP^+^ and/or magnolol on mitochondrial REDOX activity in human neuroblastoma SH-SY5Y cells. Cells were treated with 0.5–5.0 mM MPP^+^ for 24 h (a). Cells were treated with 2.5 mM MPP^+^ in the presence or absence of magnolol (1 or 3 *μ*M) for 24 h (b). Mitochondrial REDOX activity was assessed by the Alamar blue assay. Alamar blue was added and cells were incubated for 120 min. These are representative results from at least three independent experiments. Results are means ± SD (*n* = 4). Statistical comparisons were carried out by one-way ANOVA followed by Dunnett's test. **P < 0.01 versus control or vehicle (DMSO), ^#^ *P* < 0.05 versus MPP^+^ + vehicle (DMSO).

To investigate the action of MPP^+^, a toxic metabolite of MPTP, on mitochondrial REDOX activity, human neuroblastoma SH-SY5Y cells were treated with various concentration of MPP^+^ (0.5–5.0 mM) for 24 h. Mitochondrial REDOX activity, as an indicator of cytotoxicity, was assessed by the Alamar blue fluorescence. MPP^+^-induced cytotoxicity showed the concentration-dependent manner (Figure 5(a)). But we confirmed that most of the cells detached from the plate with the concentration of 5.0 mM (data not shown). Therefore, the concentration of 2.5 mM MPP^+^ was chosen for the induction of decrease in mitochondrial REDOX activity with the incubation time of 24 h. To examine the effect of magnolol on MPP^+^-induced cytotoxicity, cells were treated to 2.5 mM MPP^+^ in the presence or absence of magnolol (1 or 3 *μ*M) for 24 h (Figure 5(b)). Mitochondrial REDOX activity decreased to 67% of control by 2.5 mM MPP^+^ treatment. Treatment with 1 or 3 *μ*M magnolol significantly attenuated MPP^+^-induced decrease to 85% or 83% of control, respectively. Magnolol alone did not affect mitochondrial REDOX activity.

### 3.4. Effect of Magnolol on MPP^+^-Induced ROS Production

**Figure 6:**
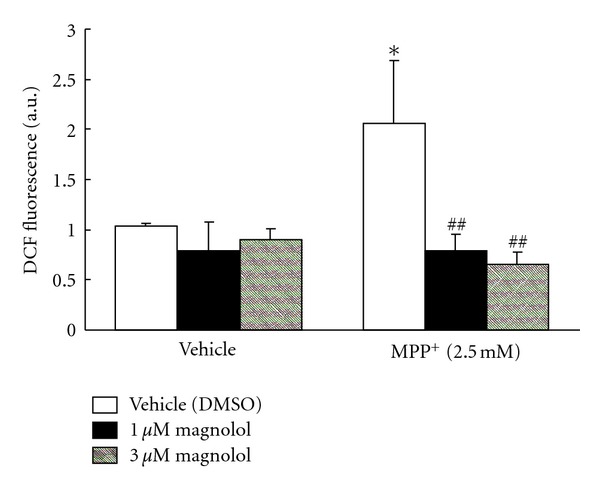
Effect of magnolol on ROS production in MPP^+^-treated human neuroblastoma SH-SY5Y cells. Cells were incubated with H_2_DCF-DA for 30 min at 37°C. And then, 2.5 mM MPP^+^ in the presence or absence of magnolol (1 or 3 *μ*M) were exposed for 24 h. ROS production was assessed by DCF fluorescence. These are representative results from two independent experiments. Results are means ± SD (*n* = 3). Statistical comparisons were carried out by one-way ANOVA followed by Dunnett's test. *P < 0.05 versus vehicle (DMSO), ^##^ *P* < 0.01 versus MPP^+^ + vehicle (DMSO).

The protective effect of magnolol on MPP^+^-induced cytotoxicity may be the result of attenuating the ROS production. To examine this possibility, cells were treated with magnolol and MPP^+^ for 24 h, and ROS production was measured by using the fluorescent dye H_2_DCF-DA (Figure 6). MPP^+^ treatment led to a 2 fold increase in DCF fluorescence compared with vehicle treatment. Treatment with magnolol (1 or 3 *μ*M) almost completely inhibited MPP^+^-induced ROS production. Magnolol alone did not affect ROS production.

## 4. Discussion

In the present study, *in vivo* and *in vitro* models of PD were used to examine the neuroprotective properties of magnolol. We first evaluated the oral administration of magnolol effectively protects striatal dopaminergic neurons in the MPTP-treated mouse model of PD. Magnolol is rapidly absorbed from the gastrointestinal tract after oral administration and peaks in the blood within 15 min of administration [27]. Moreover, it is rapidly accumulated in the various regions of brain after intravenous administration [28]. Therefore, we presume that oral administered magnolol is absorbed from the gastrointestinal tract, cross the blood-brain barrier, accumulated and act on the brain including striatum of mice.

Our results demonstrated that both pre- and post-MPTP treatment with magnolol significantly prevented MPTP-induced decrease in DAT and TH protein levels in the striatum (Figures 2(b) and 2(c), 3(b), and 3(c)). However, both treatments with magnolol did not attenuate MPTP-induced increase in GFAP levels (Figures 2(d) and 3(d)). The reason for this discrepancy is still unknown. GFAP is well known to be a marker for reactive astrocytes in the response to central nervous system injury [29]. Magnolol may prevent the dopaminergic neuron associated with the activation of astrocytes in MPTP-induced neurotoxicity, however it did not affect GFAP levels in normal condition. The several studies have shown that astrocytes can confer neuronal protection by synthesizing and releasing the free-radical scavenger glutathione [30, 31]. In addition, the activated astrocytes may stimulate microglial cells, which induce dopaminergic sprouting via the synthesis of neurotrophic factors [32]. Moreover, we evaluated the effect of magnolol against MPTP-induced oxidative stress in an *in vivo* model. At 6 h after MPTP treatment, dopaminergic neurodegeneration was not identified in the striatum, because DAT, TH, and GFAP protein levels as determined by western blot analysis were not changed in the striatum as compared with that of control mice (data not shown). We found that MPTP treatment induces the increase in lipid peroxidation product TBARS levels before the loss of dopaminergic neurons, and magnolol inhibit the TBARS production (Figure 4). It is well known that magnolol has antioxidant and free-radical scavenging activities [33–35]. These findings indicate that oral administration of magnolol prevents MPTP-induced dopaminergic neurodegeneration, and its protective effect could be, at least in part, associated with the attenuation of MPTP-induced oxidative stress. It is unlikely that the protective effects of magnolol are mediated through inhibition of monoamine oxidase-B (MAO-B), because magnolol is inactive against both types of MAO-A and MAO-B from rat brain mitochondria [36].

In order to investigate whether magnolol could directly protect against MPP^+^-induced cytotoxicity, we used an *in vitro* system human neuroblastoma SH-SY5Y cells. Alamar blue is a fluorescent indicator that is intermediate only between final reduction of molecular oxygen and cytochrome oxidase. Therefore, the REDOX potential of Alamar blue allows for the respiratory chain to function to near completion, which will provide a more accurate and sensitive indication of mitochondrial function. Mitochondrial REDOX activity was observed to decline after MPP^+^ treatment, and treatment with magnolol reduced this decline (Figure 5(b)). In addition, we confirmed that magnolol improved MPP^+^-induced morphological changes observed during apoptosis in the neuroblastoma (data not shown). ROS production was found to increase after MPP^+^ treatment, and treatment with magnolol suppressed significantly MPP^+^-induced ROS production (Figure 6). These results indicate that magnolol protects directly the neuroblastoma against the toxic-insult through the antioxidative activity.

The neurotoxic effects of MPTP are thought to be mediated by its metabolite MPP^+^ which is caused by the oxidation of MPTP by MAO-B in glial cells [37]. MPP^+^ is taken up by dopaminergic neurons via DAT as it has a high affinity for this transporter [38]. Several *in vitro* studies have suggested that MPP^+^ is concentrated within mitochondria [39] and inhibits mitochondria complex I in the mitochondrial respiratory chain [40]. This damage can lead to a number of deleterious effects on mitochondrial function, including the loss of mitochondrial membrane potential and inhibition of mitochondrial ATP production [41, 42]. In addition, inhibition of complex I increases the production of ROS, which impairs the electron transport and ATP production [11, 12, 43]. Therefore, the observed MPP^+^-induced decrease in the mitochondrial REDOX activity may reflect the extent of mitochondrial damage due to increase of ROS production. We have found that magnolol was effectively preserving mitochondrial REDOX activity, perhaps at least in part, through anti-oxidative effect. However, the mechanism is not fully understood whether magnolol improves MPP^+^-induced mitochondrial failure such as decrease in membrane potential. Further studies are needed to investigate the precise protective mechanism of magnolol against MPTP/MPP^+^-induced cytotoxicity.

Recent report has shown that subchronic treatment with magnolol significantly ameliorates apomorphine-induced contralateral rotation in 6-OHDA-treated mice and prevents 6-OHDA-induced decrease in TH protein levels in the striatum [21]. Because 6-OHDA-induced toxicity is relatively selective for catecholaminergic neurons, neurotoxin to is widely used produce PD models [44, 45]. The mechanisms underlying toxicity of 6-OHDA is involved in the production of several toxic oxidative species including superoxide radicals, hydrogen peroxide, and hydroxyl radicals [46, 47]. Therefore, magnolol has exhibited neuroprotective effect in other PD mouse model via perhaps its antioxidative properties.

Another mechanism that may explain the protective effect of magnolol in preventing MPTP/MPP^+^-induced cytotoxicity is the regulation of neurotrophic effect. It has been reported that magnolol and honokiol promote the neurite outgrowth and enhance the neuronal survival through these neurotrophic effects in cultured rat cortical neurons [22]. In 6-month-old SAMP8 mice, 14-day treatment with magnolol (10 mg/kg) at 2 months of age prevented an age-related decrease in the density of cholinergic neurons mediated the increase of Akt phosphorylation in the forebrain [20]. Akt pathway is well known as a prominent regulator of neurotrophin-mediated survival responses in neurons [48]. Several lines of evidence indicated that the Akt signaling pathway responds to oxidative stress, and the promotion of Akt phosphorylation exerts a neuroprotective effects in the model of PD [49, 50].

In conclusion, our results clearly demonstrate that magnolol protects against MPTP/MPP^+^-induced toxicity in both *in vivo* and *in vitro* models of PD. The protective mechanism could be partly associated with its anti-oxidative effect. Magnolol may be a useful therapeutic agent for the neuroprotective treatment of idiopathic PD.
